# Stroke Increases Neural Stem Cells and Angiogenesis in the Neurogenic Niche of the Adult Mouse

**DOI:** 10.1371/journal.pone.0113972

**Published:** 2014-12-01

**Authors:** Rui Lan Zhang, Michael Chopp, Cynthia Roberts, Xianshuang Liu, Min Wei, Siamak P. Nejad-Davarani, Xinli Wang, Zheng Gang Zhang

**Affiliations:** 1 Department of Neurology, Henry Ford Hospital, Detroit, Michigan, United States of America; 2 Department of Physics, Oakland University, Rochester, Michigan, United States of America; School of Pharmacy, Texas Tech University HSC, United States of America

## Abstract

The unique cellular and vascular architecture of the adult ventricular-subventricular zone (V/SVZ) neurogenic niche plays an important role in regulating neural stem cell function. However, the in vivo identification of neural stem cells and their relationship to blood vessels within this niche in response to stroke remain largely unknown. Using whole-mount preparation of the lateral ventricle wall, we examined the architecture of neural stem cells and blood vessels in the V/SVZ of adult mouse over the course of 3 months after onset of focal cerebral ischemia. Stroke substantially increased the number of glial fibrillary acidic protein (GFAP) positive neural stem cells that are in contact with the cerebrospinal fluid (CSF) via their apical processes at the center of pinwheel structures formed by ependymal cells residing in the lateral ventricle. Long basal processes of these cells extended to blood vessels beneath the ependymal layer. Moreover, stroke increased V/SVZ endothelial cell proliferation from 2% in non-ischemic mice to 12 and 15% at 7 and 14 days after stroke, respectively. Vascular volume in the V/SVZ was augmented from 3% of the total volume prior to stroke to 6% at 90 days after stroke. Stroke-increased angiogenesis was closely associated with neuroblasts that expanded to nearly encompass the entire lateral ventricular wall in the V/SVZ. These data indicate that stroke induces long-term alterations of the neural stem cell and vascular architecture of the adult V/SVZ neurogenic niche. These post-stroke structural changes may provide insight into neural stem cell mediation of stroke-induced neurogenesis through the interaction of neural stem cells with proteins in the CSF and their sub-ependymal neurovascular interaction.

## Introduction

The ventricular-subventricular zone (V/SVZ) of the lateral ventricles in adult rodent brain is a neurogenic niche which contains neural stem cells that generate intermediate neural progenitor cells. These intermediate neural progenitor cells, in turn, differentiate into olfactory bulb interneurons throughout animal’s life [1]–[4]. Stroke increases neurogenesis and newly generated neuroblasts in the V/SVZ migrate to the ischemic boundary region [5]–[7]. These cells are required for brain repair and functional recovery after stroke, since the ablation of neuroblasts after stroke substantially enlarges infarction and exacerbates neurological outcome [8]. Previously, we reported that stroke-increased neuroblasts were rapidly generated after elimination of actively dividing intermediate neural progenitor cells in the V/SVZ by an anti-mitotic drug, suggesting that slowly dividing neural stem cells contribute to stroke-induced neurogenesis [9]. However, due to technical challenges of identifying neural stem cells in the V/SVZ there is no direct in vivo evidence demonstrating the effect of stroke on neural stem cells.

Neurogenesis couples to angiogenesis in ischemic brain [10], [11]. Stroke-induced new blood vessels in ischemic boundary region provide scaffolds to guide neuroblasts to the region [10], [11]. Moreover, activated cerebral endothelial cells in angiogenic vessels secrete cytokines to attract neuroblasts [12]. The blockage of stroke-induced angiogenesis reduces neurogenesis [10]. Cerebral blood vessels in the V/SVZ niche exhibit a planar vascular plexus that is distinct from the tortuous morphology of cerebral vessels in non-neurogenic regions [13], [14]. Under physiological conditions, neural stem cells and intermediate neural progenitor cells directly contact V/SVZ planar vessels [13], [14]. Little is known about changes of neural stem cells and vascular architecture within the V/SVZ neurogenic niche in response to stroke.

Using whole-mount preparation of the lateral ventricle wall, recent studies have revealed that within the V/SVZ niche, slowly dividing glial fibrillary acidic protein (GFAP)-positive neural stem cells bridge the ventricle and the blood vessels in the SVZ by their apical single cilium to directly contact the cerebrospinal fluid (CSF), and by their long basal processes to reach blood vessels, respectively [1] [13], [14]. The unique cellular and vascular architecture of the V/SVZ niche plays an important role in regulating neural stem cell function through interaction with extracellular matrix (ECM) proteins and/or neurovascular interaction under physiological conditions [13]–[15]. In the present study, we capitalize on the whole-mount preparation of the lateral ventricle wall to examine the architecture of neural stem cells and blood vessels in the V/SVZ over a course of 3 months after onset of stroke. We found that stroke considerably altered the architecture of the V/SVZ neurogenic niche of the adult mouse by augmentation of neural stem cells and cerebral blood vessels.

## Materials and Methods

All experimental procedures have been approved by the Institutional Animals Care and Use Committee of Henry Ford Hospital.

### Animal model of stroke

For this study, we used young-adult (3 months) wild-type male C57/BL6 (n = 47) (Jackson Laboratory) and age-matched doublecortin (DCX) enhanced green fluorescent protein transgenic male mice (n = 21) (DCX-eGFP/bacterial artificial chromosome, catalog 000244-MU, the Mutant Mouse Regional Resource Center). The right middle cerebral artery (MCA) was permanently occluded by placement of a filament at the origin of the MCA [16]–[18]. Briefly, under the operating microscope (Carl Zeiss, Inc.), the right common carotid artery (CCA), the right external carotid artery (ECA) and the internal carotid artery (ICA) were isolated via a midline incision. A 6-0 nylon filament with an expanded tip was gently advanced from the ECA into the lumen of the ICA. The tip of the filament was positioned at the origin of the MCA [16], [17]. Ischemic mice were sacrificed at various time points after MCA occlusion (MCAO). In this model of MCAO, the ischemic lesion does not encompass the SVZ and stroke induces neural progenitor cell proliferation in the V/SVZ [16], [17]. Our unpublished data show that in this mouse model sham operation does not affect SVZ neurogenesis compared to non-operated animals. Therefore, mice without any surgery were used as a control group. The condition of the mice, particularly hydration levels, was closely monitored within the first week of MCAO. If the mice became dehydrated, fluid was administered by subcutaneous injection.

#### Bromodeoxyuridine (BrdU) labeling

BrdU, the thymidine analog that is incorporated into the DNA of dividing cells during S-phase, was used for mitotic labeling (Sigma Chemical). To identify slowly dividing neural stem cells, mice intraperitoneally (i.p) injected with BrdU (50 mg/kg) daily for 5 consecutive days starting at the day of MCAO and animals were sacrificed 30 days after MCAO. To detect proliferating endothelial cells, mice were treated with BrdU (50 mg/kg, i.p) every 2 h for three times and sacrificed 1 h after last injection.

### Whole-Mount Preparation

Whole-mount preparation of the lateral wall of the lateral ventricle (LV) was performed according to the published protocol [1], [19]. Briefly, following anesthetization, the mouse was transcardially perfused with heparinized saline. The brain was extracted. The LV was dissected from the caudal aspect of the telencephalon, and the hippocampus and septum were removed. The dissection was included the majority of the lateral wall [19]. The dissected lateral wall was fixed in 4% paraformaldehyde with 0.1% Triton-X 100 at 4°C overnight for immunohistochemitry.

### Immunohistochemistry and imaging

Whole-mount immunostaining was performed according to published protocols [1], [19]. Briefly, fixed whole mounts were washed in phosphate buffered saline (PBS) with 0.1% Triton-X 100 and then incubated with primary antibodies at 4°C for 48 h. After that, whole mounts were incubated with secondary antibodies for an additional 48 h. The following primary antibodies were used in the present studies: mouse anti-acetylated tubulin (1∶1000, Sigma-Aldrich), rabbit anti-γ-tubulin (1∶1000, Sigma-Aldrich), mouse anti-β-catenin (1∶200, BD Transduction Labs), rabbit anti-β-catenin (1∶200, Spring Bioscience), mouse anti-GFAP (1∶500, Chemicon), rabbit anti-GFAP (1∶5000; Dako), rabbit anti-phosphorylated histone H3 (PH3, 1∶500, Upstate), goat anti-doublecortin (DCX, 1∶200, Santa Cruz Biotechnology), rabbit anti-Ki67 (1∶200, Lab Vision), rat-anti-BrdU (1∶200, Accurate Chemicals), rabbit anti-collagen IV (1∶500, Abcam), and rat anti-CD31 (1∶50, BD Bioscience). The primary antibodies were visualized by fluorescein isothiocyanate (FITC)-, Cy3-, or Cy5-conjugated secondary antibodies (Jackson Laboratory).

Three dimensional images were acquired using Zeiss two-photon microscopy (Zeiss LSM 510 NLO) [17], [20]. The images were taken from the apical surface down to the basal whole mount with 0.5 or 1 µm interval at Z axis under a 40× or 63× objective, with total 20 or 50 µm in thickness. Non-overlap fields (9–12) were acquired from anterior-dorsal (AD), anterior-ventral (AV) and posterior dorsal (PD) of a whole mount [1], [19]. The density of immunoreactive cells in each image was measured according to our published protocol [17], [20]. The data are presented as the average of imaged fields. Cerebral vascular structures in images with 50 µm thickness were measured using our home-made software, 3-D vessel quantification program [21], [22]. Briefly, the length of each branch was determined by counting the number of skeleton voxels in that branch. The diameter of each vascular branch was computed by examining the voxels in the direction normal to the medial axis. Vascular diameters of ≤7.5 µm were defined as capillaries, while diameters between >7.5 µm and ≤30 µm were considered as venules and arterioles [22]. Four to five whole mount samples were used for each group.

#### Statistical analysis

All data are presented as mean ± SE. Significant differences between two groups were analyzed using student’s t test. Statistical significance was set at *p*<0.05.

## Results

### Stroke increases neural stem cells in the SVZ

The mortality rate for wild-type mice and eGFP-DCX transgenic mice was 11% and 13%, respectively.

**Figure 1:**
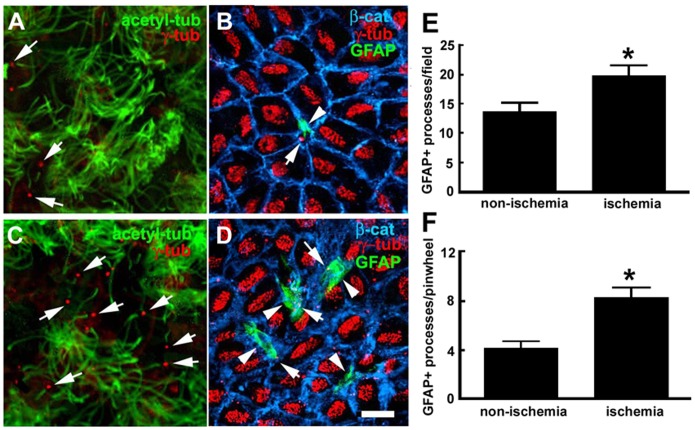
GFAP^+^ processes at ventricular surface. Double and triple immunofluorescent images acquired from the apical surface of representative non-ischemic (A, B) and ischemic (C, D) whole mounts show acetylated tubulin^+^ cilium and γ-tubulin^+^ basal bodies (A, C) and cells with single γ-tubulin^+^ basal body, GFAP^+^ processes at the center of β-catenin^+^ cobblestone ependymal cells (B, D). Quantitative data (E, F) show the number of cells with GFAP^+^ processes on the apical surface. *p<0.05, n = 6 mice/group. Bar = 10 µm.

**Figure 2:**
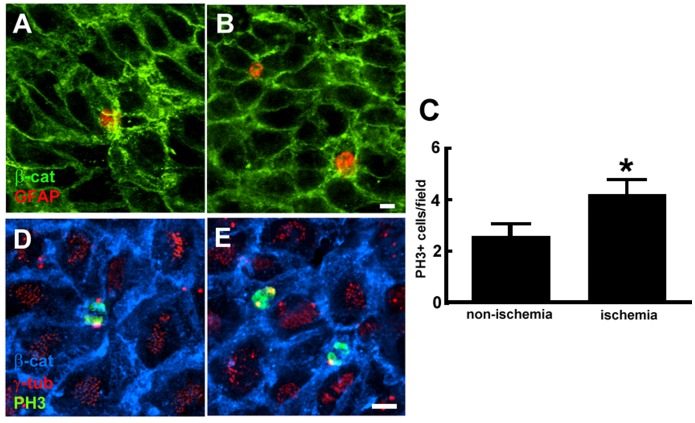
BrdU^+^ nuclei at the center of pinwheel structure. Double and triple immunofluorescent images acquired from just below the apical surface of representative non-ischemic (A, D) and ischemic (B, E) whole mounts show BrdU^+^ nuclei (A, B) and PH3^+^ nuclei with single γ-tubulin^+^ basal body (D, E) at center of β-catenin^+^ ependymal cells. Quantitative data (C) show the number of cells with BrdU^+^ and PH3^+^ nuclei. *p<0.05, n = 6 mice/group. Bar = 10 µm.

Using the lateral ventricular wall whole-mount preparations [1], [19], we examined neural stem cells in the V/SVZ niche under non-ischemic and ischemic conditions. Three dimensional analysis of whole-mount tissue with confocal microscopy was primarily in anterior-dorsal (AD), anterior-ventral (AV), and posterior-dorsal (PD) regions of the ventricular wall which contain the highest number of neural stem cells [1], [19]. In non-ischemic whole mounts, double immunofluorescent staining showed the presence of ependymal cells identified by multiple γ-tubulin^+^ basal bodies and multiple long acetylated tubulin^+^ cilia on the ventricular surface (Fig. 1). Cells with single γ-tubulin^+^ basal body with single short acetylated tubulin^+^ cilium were also evident (Fig. 1). Triple immunofluorescent staining revealed that cells with single γ-tubulin^+^ basal body on the surface of the ventricular wall were GFAP^+^ and surrounded by β-catenin^+^ cobblestone ependymal cells resembling as a pinwheel (Fig. 1). These are adult neural stem cells in the V/SVZ niche [1]. We found that whole-mounts from mice subjected to 30 days of MCAO showed an increase in the number of single γ-tubulin^+^ basal body with single short acetylated tubulin^+^ cilium and the number of GFAP^+^ cells with single γ-tubulin^+^ basal body on the ventricular wall (Fig. 1A–F)). To further verify neural stem cells, we used a label-retaining approach in which we injected BrdU to mice daily for 5 consecutive days starting on the day of MCAO. These mice were sacrificed 30 days after MCAO, i.e.25 days after the last injection, based on the fact that adult neural stem cells are slowly dividing [14]. Stroke increased the number of BrdU^+^ cells at the center of pinwheel structure (Fig. 2). A pair of BrdU^+^ nuclei were frequently observed in ischemic whole mounts (Fig. 2), suggesting that these cells were actively proliferating. Indeed, triple immunofluorescent staining with antibodies against PH3, a marker of the G_2_/M phases of proliferating cells [23], revealed that stroke increased PH3+ cells with single γ-tubulin^+^ basal body at the center of β-catenin^+^ pinwheel structure and these cells were in the mitotic telophase as evident by a pair of PH3^+^ nuclei (Fig. 2). None of β-catenin^+^ cobblestone ependymal cells were BrdU^+^. Collectively, these data indicate that stroke increases neural stem cells.

### Stroke induces V/SVZ angiogenesis

**Figure 3:**
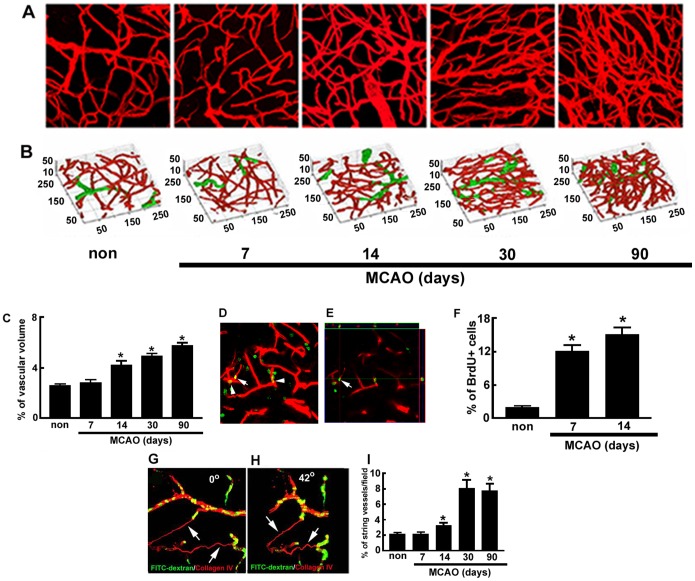
Cerebral microvasculature in the V/SVZ. Original composite (A) and corresponding three-dimensional (B) images of collagen IV^+^ cerebral blood vessels from representative non-ischemic whole mount (non), and 7, 14, 30, and 90 days of ischemic whole mounts. Red and green colors in panel B represent diameters of blood vessels less than 7.5 µm and larger than 7.5 µm, respectively. Panel C shows quantitative data of blood vessel volume. The data were generated by the three-dimensional vessel quantification program. Unit for numbers in all images is microns. Representative double immunofluorescent images (D, E) show that CD31^+^ cells (red) were BrdU^+^ (green, arrow and arrowheads) at a composite view (D) and that an orthogonal view (E) revealed co-localization of a CD31+/BrdU+ cell shown in the panel D (arrow). Panel F shows quantitative data of CD31^+^/BrdU^+^ cells. Representative three dimensional images (G, H) show views of collagen IV^+^ string vessels (red, arrows) that were not perfused by FITC-dextran (green) from 0 (G) and 46 (H) degree angles. Panel I shows quantitative data of sting vessels over 90 days of MCAO. *p<0.05 vs non-ischemic group, n = 6 mice/group.

The V/SVZ contains a unique planar vascular plexus [13], [14]. To examine the effect of stroke on V/SVZ blood vessels, we imaged microvascular structures in the AD, AV, and PD regions of whole-mount preparations obtained from mice sacrificed from 7 to 90 days after MCAO. Collagen IV immunoreactive vessels in these regions were three-dimensionally imaged starting from the ventricular surface and down to the SVZ by means of confocal microscopy. Consistent with published studies, 3D image analysis revealed that the V/SVZ niche had a planar vascular plexus and the majority of blood vessels were capillaries in non-ischemic mice (Fig. 3). However, stroke considerably increased blood vessels starting 14 days after MCAO (Fig. 3). Quantitative analysis of vascular volume showed that in non-ischemic V/SVZ, blood vessels constituted 2.6% of the total volume, which is comparable with published data showing that the vascular density in the SVZ is 2.5% [24]. After stroke, blood vessels increased to 4.2, 4.9, and 5.7% at 14, 30 and 90 days, respectively (Fig. 3). The majority of increased blood vessels were capillaries (Fig. 3).

To examine whether increased blood vessels are related to augmentation of endothelial cell proliferation, we quantified the number of CD31^+^ endothelial cells which were BrdU^+^. Only 2% of CD31^+^/BrdU^+^ cells were detected in non-ischemic whole-mount tissues. However, CD31^+^/BrdU^+^ cells increased to 12 and 14% at 7 and 14 days after stroke, respectively (Fig. 3). Co-localization of CD31 and BrdU immunoreactivity was confirmed by 3D images (Fig. 3). These data along with increased blood vascular volume indicate that stroke induces angiogenesis in the V/SVZ niche.

Strikingly, an increase in blood vascular volume was associated with substantial increases of string vessels over the same period (Fig. 3). To examine whether the string vessel carries blood, we intravenously administered FITC-dextran before the mice were sacrificed. FITC-dextran circulates along with plasma within blood vessels. We found that string vessels were not perfused by FITC- dextran (Fig. 3), indicating that they are not functional vessels. BrdU+ endothelial cells within the string vessels were not detected.

### Association of stroke-induced neurogenesis and angiogenesis

**Figure 4:**
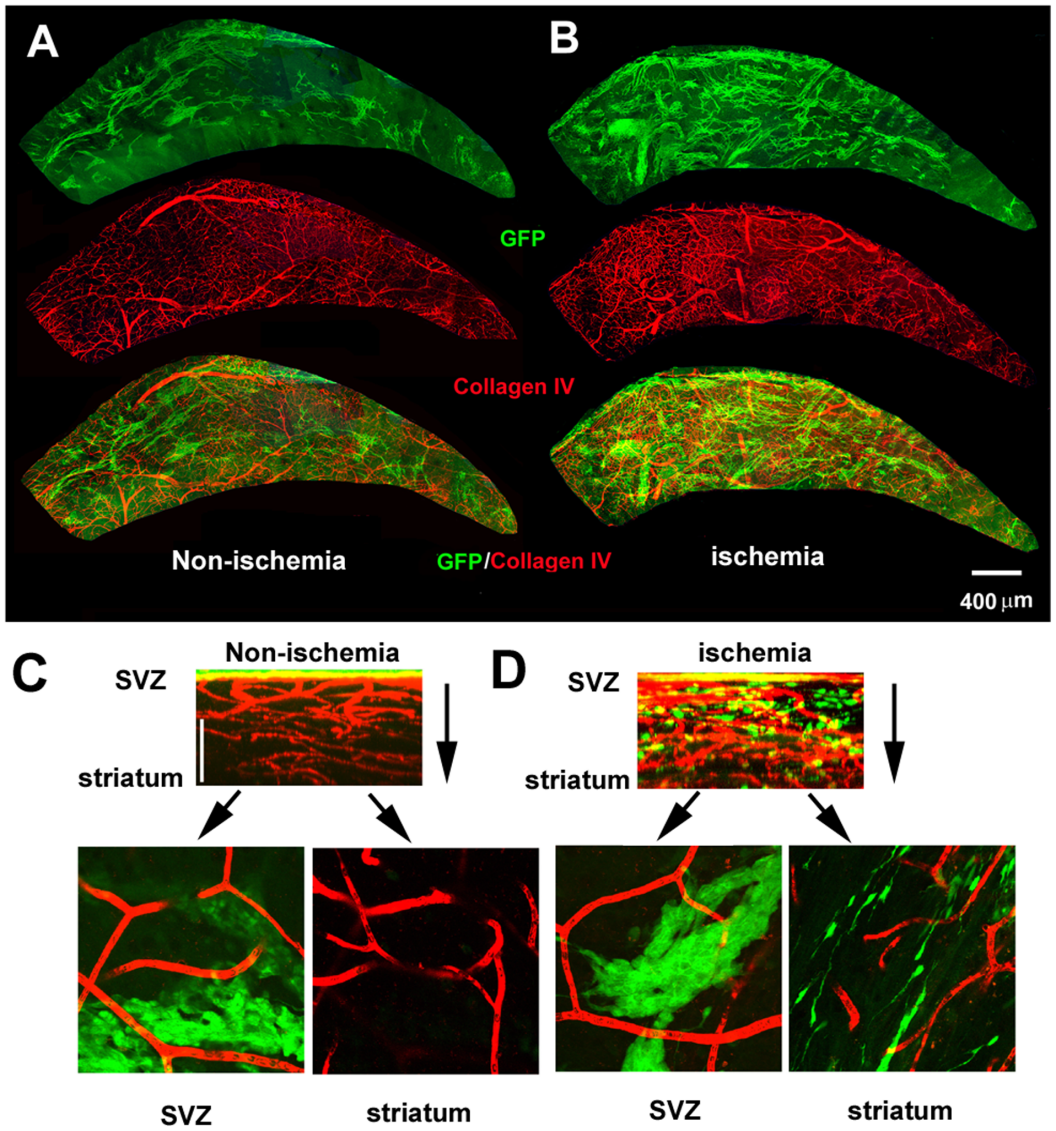
Microvascular structure and neuroblasts in the V/SVZ. Double immunofluorescent images acquired from representative non-ischemic (A) and ischemic (B) whole mounts (viewed from the ventricular surface) show the network of collagen IV^+^ blood vessels (red) and distribution of GFP^+^ neuroblasts (green). Projected images with total thickness of 50 µm from the Z axis (C, D) show that GFP^+^ neuroblasts in non-ischemic whole mount were restricted in the SVZ (C, green), while GFP^+^ neuroblasts in ischemic whole mount distributed from the SVZ (D, green) down to striatum (D, green). Single composite image with 1 µm thickness from X–Y axis at SVZ and striatum beneath the ventricular surface from their corresponding Z stacks (C, D) show collagen IV^+^ blood vessels and GFP^+^ neuroblasts.

Using DCX-eGFP transgenic mice, we previously demonstrated that migration of neuroblasts out of the V/SVZ is closely associated with blood vessels in ischemic striatum [11]. To examine association of neuroblasts and blood vessels in the V/SVZ after stroke, we prepared whole mounts of the lateral wall of the lateral ventricle of DCX-eGFP transgenic mice subjected to 30 days of MCAO. 3D images were acquired from the ventricular surface down to the striatum of the entire whole mount that was double immunofluorescently stained with antibodies against collagen IV and GFP for indentifying cerebral blood vessels and neuroblasts, respectively. In non-ischemic whole mounts, assembled montage reconstructions revealed the presence of the vascular plexus with large vessels running from the dorsal and ventral peripheries, which branched into small vessels (Fig. 4). The GFP^+^ neuroblasts were distributed within the lateral well of the LV with high density occurring in the anterior dorsal region of the whole mount, which ran parallel to large blood vessels (Fig. 4). 3D images projected from the Z axis of the ventricular surface down to the striatum showed that neuroblasts were restricted to the SVZ. These findings are consistent with published studies [11], [13], [14], [25]. However, stroke substantially increased the number of large and small blood vessels, and GFP^+^ neuroblasts expanded from neurogenic regions to nearly the entire of the lateral ventricular wall (Fig. 4). Increased blood vessels and neuroblasts also extended from the ventricular surface to the striatum (Fig. 4). We did not observe significant changes of the lateral ventricular surface area between ischemic and non-ischemic mice.

**Figure 5:**
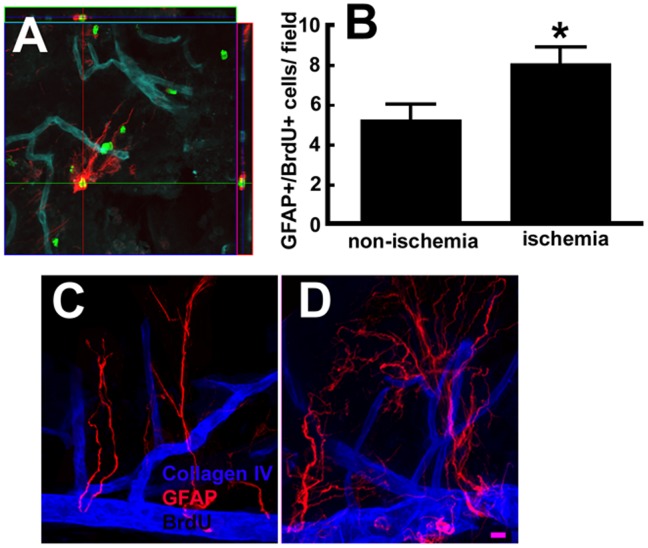
GFAP^+^/BrdU^+^ cells and blood vessels in the V/SVZ. A representative orthogonal view of triple immunofluorescent image (A) shows that a BrdU^+^ (green) and GFAP^+^ cell with long processes (red) contacted with collagen IV^+^ blood vessels (blue). Panel B shows quantitative data of BrdU^+^/GFAP^+^ cells. Double immunofluorescent images from representative non-ischemic (C) and ischemic (D) whole mounts show GFAP^+^ long processes contacted collagen IV^+^ blood vessels.

Physiologically, GFAP^+^/BrdU^+^ neural stem cells and actively dividing intermediate neural progenitor cells in the V/SVZ are in close proximity to the blood vessels [13], [14]. We thus assessed the association of neural stem cells with blood vessels in the V/SVZ niche of wild-type mice subjected to the BrdU label-retaining approach and sacrificed 30 days after MCAO. Measurements were performed in the regions of AD and PD of the whole-mount tissues. Consistent with published studies by others [1], [19] in non-ischemic whole-mount tissues we found that GFAP^+^/BrdU^+^ cells had a few GFAP+ processes (Fig. 5) which are distinct from the multipolar process morphology of parenchymal and reactive astrocytes [14]. GFAP^+^ long processes in the V/SVZ niche made contact with blood vessels (Fig. 5). Stroke substantially increased these GFAP^+^ long processes (Fig. 5), suggesting that neural stem cells sense changes in signals from a network of angiogenic vessels after stroke.

## Discussion

Using whole-mount preparations of the lateral ventricle wall, the present study examined changes of cellular and vascular architecture in V/SVZ niche in response to focal cerebral ischemia. Stroke induced-neurogenesis has been demonstrated in experimental animals and human patients [5]–[7]; however, there is no direct in vivo evidence showing the effect of stroke on neural stem cells and their relationship with blood vessels in the V/SVZ of the adult rodent. We show that stroke considerably increased GFAP+ neural stem cells at the center of the pinwheel structure composed of ependymal cells, and that their GFAP+ elongated processes were in direct contact with augmented-blood vessels just beneath the ependymal layer in the V/SVZ niche. These data suggest that neural stem cells are actively involved in stroke-induced neurogenesis by interacting with the CSF and the vasculature in the V/SVZ niche.

Adult neural stem cells in the V/SVZ niches are a subpopulation of astrocytes that are slowly dividing [1]. These neural stem cells generate actively proliferating intermediate neural progenitor cells that in turn to give rise to migrating neuroblasts [26], [27]. We previously reported that stroke-increased neuroblasts were rapidly generated after eliminating actively dividing neural progenitor cells in the V/SVZ niche by an anti-mitotic drug, which suggests that neural stem cells in the V/SVZ contribute to stroke-induced neurogenesis [9]. We now provide direct evidence showing that stroke substantially increased neural stem cells in the V/SVZ niche. Based on morphology and phenotypes of adult neural stem cells established by Mirzadeh et al [1], the present study showed that stroke substantially increased stem cell astrocytes localized at the center of pinwheels. Moreover, the BrdU label-retention approach indicates that stem cell astrocytes are actively proliferating. The SVZ also contains nongerminal astrocytes (B2 cells) that are mainly localized at the SVZ-striatal border, 30–40 µm beneath the ventricular surface [1], [13], [14]. The B2 astrocytes have multipolar process morphology and contact blood vessels with their foot-processes [13], [14]. Astrocytes activated by stroke have multiple thick GFAP^+^ processes [25], [28], [29]. In the present study, to minimize overlapping stem cell astrocytes (B1 cells) with nongerminal astrocytes (B2 cells), we only measured GFAP^+^/BrdU^+^ astrocytes with a few processes within 20 µm beneath the ependymal layer. Collectively, these data suggest that quiescent adult neural stem cells in the V/SVZ niche can be recruited to an active pool to increase the neurogenic process in response to ischemic insult.

Using rat brain coronal sections, we previously showed that stroke induced ependymal cell proliferation based on their cellular morphology and their radial glial phenotype [20]. In the present study, using whole-mount preparations of the lateral ventricle wall in combination with molecular markers and cytoarchitecture to characterize ependymal cells, we did not find any BrdU^+^ ependymal cells on the ventricular surface beyond 30 days of stroke onset. Nevertheless, the present study showed that stroke-increased BrdU^+^ neural stem cells with a single cilium at the center of ependymal cells displayed radial glial-like morphology. These features are very difficult to distinguish from ependymal cells on the coronal thin sections, which likely misled us to consider these cells as proliferative ependymal cells in our previous study [20]. Our new data highlight the importance of employing whole-mount preparations of the lateral wall of the ventricle in combination with an immunohistochemistry approach to identify adult neural stem cells after stroke.

In the aging brain, stem cell astrocytes in the V/SVZ exhibit antigenic and morphologic characteristics of ependymal cells and mediate ependymal repair [30]. Using the whole-mount preparations of the lateral ventricle wall, Young et al reported that stroke induced ependymal cells to robustly express GFAP, which led them to conclude that stroke induces reactive astrocytosis in the V/SVZ niche [25]. As noted in their article, Young et al stated that the high levels of ependymal GFAP expression prevented reliable quantification of GFAP-positive neural stem cells in the V/SVZ niche [25]. However, as demonstrated in the current study, GFAP immunoreactivity was only detected in stem cell astrocytes at the center of pinwheel structure composed by ependymal cells under non-ischemic and ischemic conditions, although the antibody against GFAP used in the present study is the same one used by Young et al [25]. The discrepancy may be due to inter-strain differences, C57/BL6 in the present study vs 129 sv by Young et al [25].

Cerebral endothelial cells are relatively quiescent in the V/SVZ niche [13], [14]. The present study demonstrated that stroke considerably induced cerebral endothelial cell proliferation and angiogenesis in this neurogenic niche, which is consistent with published studies [25], [31]. Moreover, the present study showed that the increased angiogenesis was accompanied by substantial augmentation of string vessels, which are thin connective tissue strands of capillary remnants [32]. Increases in string vessels have been detected in human fetal brains when brain angiogenesis occurs [33] and in human brains with Alzheimer’s disease [32]. Radiation reduces capillary density and increases string vessels [32]. These data suggest that both damage of blood endothelial cells and angiogenesis could induce string vessel formation. Our data show that increases in string vessels were associated with augmentation of blood vessels. We thus speculate that increases in string vessels may imply a reduction in newly generated vessels. The V/SVZ contains fractones that consist of stems terminating in bulbs that are localized immediately underneath the ependymal layer and the basal site of stems that attaches to blood vessels [34]. String vessels are thin strands within the capillary network [32]. Our three-dimensional imaging data showed that collagen IV immunoreactive strands were connected between FITC perfused capillaries. We therefore referred to these strands as string vessels. However, additional ultrastructural analysis is warranted to make a conclusive distinction between fractones and string vessels.

Physiologically, the importance of the unique architecture of neural stem cells in the V/SVZ niche in the regulation of stem cell function has been well demonstrated either through interaction with ECM proteins or via neurovascular interactions [1], [13]–[15]. Our previous in vitro study demonstrated that primary neural progenitor cells harvested from the V/SVZ after stroke enhance capillary tube formation by stimulating proliferation of non-ischemic cerebral endothelial cells, while ischemic endothelial cells promote normal neural progenitor cells to generate neuroblasts [12]. We speculate that these events may also take place in the ischemic V/SVZ niche based on the current observations that stroke-induced angiogenesis in the V/SVZ niche occurs parallel to an increase in neural stem cells.

The sonic hedgehog (Shh) signaling pathway acting through the primary cilia in neural stem cells is required to maintain the adult neural stem cell pool in V/SVZ [3], [4]. Intraventricular infusion of Shh enhances stroke-increased neurogenesis [35], [36]. Stroke-increased neural stem cells with primary cilia at the ventricular surface demonstrated in the present study may contribute exogenous Shh-enhanced neurogenesis.

In summary, the present study demonstrated that stroke considerably increased the number of GFAP-positive neural stem cells in the V/SVZ. The apical primary cilia of the neural stem cells were exposed to the CSF, and their long basal processes made contact with augmented-blood vessels just beneath the ependymal layer. This stem cell/vascular remodeling may provide insight into molecular mechanisms within the V/SVZ niche that mediate stroke-induced neurogenesis. Therapies targeting this neurogenic niche may facilitate neurogenesis after stroke.
